# Supplementary material for: BREAst screening Tailored for HEr (BREATHE)—A study protocol on personalised risk-based breast cancer screening programme
Source: PLoS One. 2022 Mar 31;17(3):e0265965. doi: 10.1371/journal.pone.0265965 (PMC8970365; doi:10.1371/journal.pone.0265965)
Supplement: S3 Appendix — (PDF) [file pone.0265965.s003.pdf]

BREATHE Breast Cancer Education
Participant Study ID: ___________
Age: _____________

Breast Cancer Perception

1) Have you attended breast screening?
   - ☐ Yes
   - ☐ No

2) If yes, how often do you go (or plan to go) for screening?
   - ☐ Once a year
   - ☐ Once every 2 years
   - ☐ Do not intend to go anymore
   - ☐ Others, please specify __________

3) I believe in the importance of breast cancer screening.
   - ☐ Strongly Agree
   - ☐ Agree
   - ☐ Neither Agree nor Disagree
   - ☐ Disagree
   - ☐ Strongly Disagree

Please state your level of agreement with the following sentences/questions.

4) What do you think is your chance of getting breast cancer?

Lowest			***Average***			***Highest***
1	2	3	4	5	6	7

5) Would you say you agree that there is not much you can do to lower your chances of ***getting*** breast cancer?

Strongly Disagree					***Strongly Agree***	
1	2	3	4	5	6	7

6) Would you say you agree that there is not much you can do to lower your chances of ***dying*** from breast cancer?

Strongly Disagree					***Strongly Agree***	
1	2	3	4	5	6	7

7) I do not have any family history of breast cancer, therefore I will not get breast cancer.

**Agree**	Family history is a risk factor, but it is NOT the only risk factor – one may still develop breast cancer without family history.

Disagree TRUE! An individual may still get breast cancer without a known family history of breast cancer, although having such history does increase one's risk.

8) I am still young, therefore I will not get breast cancer.

Agree Breast cancer risk increases with age. Most cases diagnosed in Singapore are of women above 40 years old but younger women may also be affected.

Disagree TRUE! Although most cases diagnosed in Singapore are of women above 40 years old and risk increases with age, younger women have been diagnosed with breast cancer too.

9) I am still young, therefore I do not need to screen for breast cancer.

Agree Younger women may also be affected by breast cancer. Women aged 40-49 may attend screening annually. If you are below 40, you are not eligible for the national breast cancer screening programme, but you should still perform breast self-examination monthly – approximately 7-10 days from the first day of your period.

Disagree TRUE! Younger women may also be affected by breast cancer. Women aged 40-49 may attend screening annually. If you are below 40, you are not eligible for the national breast cancer screening programme, but you should still perform breast self-examination monthly – approximately 7-10 days from the first day of your period.

10) I have given birth, therefore I will not get breast cancer.

Agree Although giving birth reduces the risk of developing breast cancer, it does not eliminate your risk of breast cancer. Women who gave birth to more than 3 children have been shown to have reduced long term risk of breast cancer. Having your first child after 30 years, may also contribute to increased risk of breast cancer by 2 times.

Disagree TRUE! Women who gave birth to more than 3 children have been shown to have reduced long term risk of breast cancer. Having your first child after 30 years, may also contribute to increased risk of breast cancer by 2 times.

11) I have breastfed, therefore I will not get breast cancer.

Agree Although breastfeeding can lower breast cancer risk, this does not mean one will not develop breast cancer. There may be a higher risk of developing breast cancer if breastfeeding duration is less than a year.

Disagree TRUE! Breastfeeding does reduce the risk of breast cancer, if the breastfeeding duration lasts longer than a year. There still may be a possibility of developing breast cancer due to other risk factors.

12) I can prevent myself from getting breast cancer by eating healthily, exercising, not drinking alcohol and not smoking.

Agree Healthy eating, exercising, not drinking alcohol and not smoking is beneficial to reduce risk of all cancer. However, it does not eliminate the risk. The BEST way to protect against breast cancer is through timely screening.

Disagree TRUE! While healthy eating, exercising, not drinking alcohol and not smoking is beneficial to reduce risk of all cancer in general, it does not eliminate the risk. The best way to protect against breast cancer is still through timely screening.

13) If breast cancer is detected early, chances of surviving is high.

Agree TRUE! Breast cancer detected at its early stages (i.e. Stage 0) often allows the breast to be conserved, with only removal of the lump. Such procedures also has 99% 5-year survival rate. About 70% of cases diagnosed in Singapore are at this stage. Later detection often leads to more invasive procedure such as total removal of breast (mastectomy) and more aggressive treatment such as chemotherapy. The cost for treatment at these stages are therefore higher.

Disagree About 70% of cases diagnosed in Singapore are diagnosed at the early stage. Breast cancer detected at this stage often requires a lumpectomy – a procedure with 99% 5-year survival rate, and allows for breast conservation. Later detection often leads to more invasive procedure such as total removal of breast (mastectomy) and more aggressive treatment such as chemotherapy. The cost for treatment at these stages are therefore higher.

14) Breast cancer screening is embarrassing.

Agree Yes, you will be required to disrobe for the procedure. Do not worry as the procedure will always be performed in complete privacy. All radiographers performing mammography and breast ultrasound in Singapore are women.

Disagree Some may feel uncomfortable disrobing for the procedure. Do not worry as the procedure will always be performed in complete privacy. All radiographers

performing mammography and breast ultrasound in Singapore are women.

15) Breast cancer screening is expensive.

**Agree**	Yes, mammogram can cost from S$100-S$200 in Singapore but subsidised mammogram (MediSave payable) is available at selected clinics. Singapore citizen aged 50 and above qualify for S$50 or less (S$25 for Pioneer generation; S$0 for CHAS card holder at Singapore Cancer Society Clinic @ Bishan).
**Disagree**	Mammogram cost from S$100-S$200 in Singapore but subsidised mammogram (MediSave payable) is available at selected clinics. Singapore citizen aged 50 and above qualify for S$50 or less (S$25 for Pioneer generation; S$0 for CHAS card holder at Singapore Cancer Society Clinic @ Bishan).

16) Mammogram can trigger cancer cells to grow.

**Agree**<br>**Disagree**	Mammogram only requires a small dose of radiation – approximately 1% of lifetime background radiation by the age 50, or 6 months of background radiation in Singapore. The compression from the plates does not cause cancer cells to spread. The breast tissue will be compressed approximately 5-40 seconds for each image and at least 2 images will be taken for each breast.

Only for women above 40 years, the following statements (17-19) will appear.

17) It is inconvenient for me to go for a mammogram.

**Agree**	Yes, the procedure may be time consuming – approximately 20 minutes excluding waiting time. You can now make appointments online (www.healthhub.sg) or by calling the specific clinic. Mammogram services is also offered all over Singapore (private clinics, polyclinics, private and public hospitals).
**Disagree**	Yes! Mammogram services is offered all over Singapore (private clinics, polyclinics, private and public hospitals). Appointments should be made online (www.healthhub.sg) or by calling the specific clinic. Excluding waiting time, the entire procedure takes approximately 20 minutes.

18) I must have symptoms first before I decide to go for mammogram.

**Agree**	Mammogram can detect cancers as small as a pin head, which is often too small to be felt by breast self-

examination. Often mammogram can detect breast cancer years before physical symptoms can be felt.

Disagree TRUE! Often mammogram can detect breast cancer years before physical symptoms can be felt as mammogram can detect cancers as small as a pin head.

19) I rather go for breast ultrasound because mammogram is painful.

Agree Yes, the compression from the mammogram plates can cause some discomfort. Breast ultrasound is LESS USEFUL in detecting microcalcifications seen in early stage breast cancer, and LESS USEFUL for larger breast. Therefore mammogram is still MORE USEFUL for screening.

Disagree Yes! Breast ultrasound may be less painful, but it is also LESS USEFUL for detecting microcalcifications seen in early breast cancer and LESS USEFUL for larger breast. Therefore mammogram is still MORE USEFUL for screening.

20) After knowing the above, would you be more willing to attend regular screening?

☐ Yes
☐ No

21) Referring to your answer in the last question, please provide a reason.

____
____
____
