# Supplementary material for: BREAst screening Tailored for HEr (BREATHE)—A study protocol on personalised risk-based breast cancer screening programme
Source: PLoS One. 2022 Mar 31;17(3):e0265965. doi: 10.1371/journal.pone.0265965 (PMC8970365; doi:10.1371/journal.pone.0265965)
Supplement: S4 Appendix — (PDF) [file pone.0265965.s004.pdf]

BREATHE Recruitment Experience Survey
Participant Study ID: ____________

1) How did you get to know of our study? (You may choose more than one option)
   - ☐ Posters at the polyclinic
   - ☐ Posters at the hospital
   - ☐ Pamphlets/Brochures
   - ☐ Referral from family and/or friends
   - ☐ Approached by study coordinator
   - ☐ Others. Please specify ______________

Recruitment Experience
Please help us to understand your **recruitment experience** by selecting which of the following best describes it.

2) The buccal swab did not cause discomfort.
   - ☐ Strongly Agree
   - ☐ Agree
   - ☐ Neither Agree nor Disagree
   - ☐ Disagree
   - ☐ Strongly Disagree

3) The study coordinator explained breast cancer risk classification clearly and is able to answer all of my questions.
   - ☐ Strongly Agree
   - ☐ Agree
   - ☐ Neither Agree nor Disagree
   - ☐ Disagree
   - ☐ Strongly Disagree

4) What was the TOP reason or motivation for joining this study?
   - ☐ To learn about breast cancer
   - ☐ To find out about my risk for breast cancer
   - ☐ To change my screening habits in case I have an increased risk for breast cancer
   - ☐ To inform my children about their possible increased risk for breast cancer
   - ☐ Out of curiosity
   - ☐ Out of scientific/research interest
   - ☐ Others. Please specify: _______________________

5) Overall, I am feeling _________ to receive my breast cancer risk report.

	Strongly Agree	Agree	Neither Agree nor Disagree	Disagree	Strongly Disagree
a. Excited					
b. Scared					
c. Confident					
d. Regretful					
e. Optimistic					
f. Anxious					
g. Worried					
h. Stressed out					

i. Others. Please specify: ___________________

6) I will recommend doing a breast cancer risk classification to others (family or friends).

- ☐ Strongly Agree
- ☐ Agree
- ☐ Neither Agree nor Disagree
- ☐ Disagree
- ☐ Strongly Disagree
