# Supplementary material for: BREAst screening Tailored for HEr (BREATHE)—A study protocol on personalised risk-based breast cancer screening programme
Source: PLoS One. 2022 Mar 31;17(3):e0265965. doi: 10.1371/journal.pone.0265965 (PMC8970365; doi:10.1371/journal.pone.0265965)
Supplement: S5 Appendix — (PDF) [file pone.0265965.s005.pdf]

<u>BREATHE Report Feedback Survey</u>
Participant Study ID: ____________

1) How would you rate your overall <u>health</u> during the past week?

- ☐ Very poor
- ☐ Poor
- ☐ Fairly poor
- ☐ Average
- ☐ Fairly good
- ☐ Good
- ☐ Excellent

2) How would you rate your overall <u>quality of life</u> during the past week?

- ☐ Very poor
- ☐ Poor
- ☐ Fairly poor
- ☐ Average
- ☐ Fairly good
- ☐ Good
- ☐ Excellent

In the next section, we would like to know your level of agreement for the following statements. Options range from 1 to 5, with 1 for strongly agree and 5 for strongly disagree.

3) I have a clear understanding of my breast cancer risk classification from my report.

- ☐ Strongly Agree
- ☐ Agree
- ☐ Neither Agree nor Disagree
- ☐ Disagree
- ☐ Strongly Disagree

4) I have a clear understanding of BREATHE study recommendations from my report.

- ☐ Strongly Agree
- ☐ Agree
- ☐ Neither Agree nor Disagree
- ☐ Disagree
- ☐ Strongly Disagree

5) I am confident that my breast cancer risk classification in my report is reliable.

- ☐ Strongly Agree
- ☐ Agree
- ☐ Neither Agree nor Disagree
- ☐ Disagree
- ☐ Strongly Disagree

6) Since receiving my report on breast cancer risk classification, I feel _________.

	Strongly Agree	Agree	Neither Agree nor Disagree	Disagree	Strongly Disagree
a. Relieved					
b. Happy					
c. Motivated					
d. Regretful					
e. Disbelief					
f. Anxious					
g. Worried					
h. Stressed out					

i. Others. Please specify: ____________________

After learning about my breast cancer risk classification:

7) What do you think is your chance of getting breast cancer?

Lowest			***Average***			***Highest***
1	2	3	4	5	6	7

8) Learning about my breast cancer risk classification has affected my ability to go on with my day-to-day task

- ☐ Strongly Agree
- ☐ Agree
- ☐ Neither Agree nor Disagree
- ☐ Disagree
- ☐ Strongly Disagree

9) I will make changes to my lifestyle

- ☐ Strongly Agree
- ☐ Agree
- ☐ Neither Agree nor Disagree
- ☐ Disagree
- ☐ Strongly Disagree

10) I will make changes to my screening habits

- ☐ Strongly Agree
- ☐ Agree
- ☐ Neither Agree nor Disagree
- ☐ Disagree
- ☐ Strongly Disagree

11) To reduce my risk of breast cancer, I would like to know more about: (Select all that apply)
   - ☐ Diet to manage risk
   - ☐ Exercise to manage risk
   - ☐ Therapy options
   - ☐ Surgery options
   - ☐ Other testing options

12) With whom will you discuss your breast cancer risk classification? (Select all that apply)
   - ☐ Family members
   - ☐ Friends
   - ☐ Co-workers / colleagues
   - ☐ Primary care provider (e.g. nurse, doctor)
   - ☐ Other medical professional
   - ☐ Others. Please specify: _____________
   - ☐ Not Applicable (I do not wish to discuss it with anyone)

   If Not Applicable, proceed to question 13. If not, proceed to question 14.

13) What are the reasons for wanting to keep your breast cancer risk classification private? (Select all that apply, or none)
   - ☐ My risk profile is a private matter.
   - ☐ I don't feel that my results are important enough to share.
   - ☐ I don't think anyone is interested in my results.
   - ☐ I am concerned about how they would react to my results.
   - ☐ I may discuss my results with someone in the future, but not for now.
   - ☐ Others. Please specify: ____________________

Risk perception

In this section, we would like to know more about your perception about the use of genetics for cancer screening. Similar to before, please let us know your level of agreement for the following statements. Options range from 1 to 5, with 1 for strongly agree and 5 for strongly disagree.

14) Knowing my risk classification including my **genetic** risk for developing cancer is important.
   - ☐ Strongly Agree
   - ☐ Agree
   - ☐ Neither Agree nor Disagree
   - ☐ Disagree
   - ☐ Strongly Disagree

15) Knowing my risk classification including my **genetic** risk for cancer will motivate me to attend cancer screening according to my risk level.
   - ☐ Strongly Agree
   - ☐ Agree
   - ☐ Neither Agree nor Disagree

- ☐ Disagree
- ☐ Strongly Disagree

16) I would like to know my genetic risk classification for other health conditions, if available.
   - ☐ Strongly Agree
   - ☐ Agree
   - ☐ Neither Agree nor Disagree
   - ☐ Disagree
   - ☐ Strongly Disagree
