# Supplementary material for: BREAst screening Tailored for HEr (BREATHE)—A study protocol on personalised risk-based breast cancer screening programme
Source: PLoS One. 2022 Mar 31;17(3):e0265965. doi: 10.1371/journal.pone.0265965 (PMC8970365; doi:10.1371/journal.pone.0265965)
Supplement: S6 Appendix — (PDF) [file pone.0265965.s006.pdf]

BREATHE Satisfaction Survey
Participant Study ID: ____________

Please help us to understand your **experience** in this breast cancer screening study by answering the following questions. Please select ONE response to each question.

1) I am glad I took part in this study for breast cancer screening.
   - ☐ Strongly Agree
   - ☐ Agree
   - ☐ Neither Agree nor Disagree
   - ☐ Disagree
   - ☐ Strongly Disagree

2) I have increased my understanding about the importance of breast cancer screening through this study.
   - ☐ Strongly Agree
   - ☐ Agree
   - ☐ Neither Agree nor Disagree
   - ☐ Disagree
   - ☐ Strongly Disagree

3) I have a better understanding about my risk of developing breast cancer through this breast cancer screening study.
   - ☐ Strongly Agree
   - ☐ Agree
   - ☐ Neither Agree nor Disagree
   - ☐ Disagree
   - ☐ Strongly Disagree

4) I am more confident about breast cancer screening and its ability to pick up breast cancer.
   - ☐ Strongly Agree
   - ☐ Agree
   - ☐ Neither Agree nor Disagree
   - ☐ Disagree
   - ☐ Strongly Disagree

5) The information on breast cancer and screening shared by the study team was useful.
   - ☐ Strongly Agree
   - ☐ Agree
   - ☐ Neither Agree nor Disagree
   - ☐ Disagree
   - ☐ Strongly Disagree

6) The information on breast cancer risk management shared by the study team was sufficient.
   - ☐ Strongly Agree
   - ☐ Agree
   - ☐ Neither Agree nor Disagree

☐ Disagree
☐ Strongly Disagree

7) The reminder to attend breast cancer screening by the study team was useful.
   ☐ Strongly Agree
   ☐ Agree
   ☐ Neither Agree nor Disagree
   ☐ Disagree
   ☐ Strongly Disagree
   ☐ Not Applicable

8) What do you like about your experience in this study? (Select ALL that applies)
   ☐ Knowing my breast cancer risk
   ☐ Learning more about breast cancer
   ☐ Availability of study coordinator to answer my questions
   ☐ Guidance in booking breast cancer screening appointment
   ☐ Reminder(s) to attend breast cancer screening
   ☐ I do not like this research study. Please state your reason(s) ________________
   ☐ Others. Please specify ___________________

9) Do you intend to continue breast cancer screening? (Select ONE)
   ☐ Yes, according to the National Guideline
   ☐ Yes, but not according to the National Guideline
   ☐ No, I believe I am not at risk of developing breast cancer
   ☐ No, I am too busy
   ☐ No, mammogram screening is too expensive
   ☐ No, for other reason: __________________________________

10) If the national breast cancer screening programme is to adapt any features of BREATHE study, which do you think will be a beneficial addition? (Select ALL that applies)
   ☐ Learning of my breast cancer risk classification
   ☐ Education on breast cancer and screening by study coordinator
   ☐ Availability of a study coordinator to help navigate breast cancer screening
   ☐ Reminder(s) to attend breast cancer screening
   ☐ I do not want the national breast cancer screening programme to adapt the features of BREATHE study
   ☐ Others. Please specify ___________________

11) If the national breast cancer screening programme is to adopt a breast cancer risk classification measure similar to BREATHE, how much would you have paid for this combined assessment of your genetic risk (out-of-pocket cost)?
   ☐ S$0
   ☐ Less than S$50
   ☐ S$50 - S$99
   ☐ S$100 - S$149
   ☐ S$150 – S$199
   ☐ S$200 and above

12) Do you have any other comments/ suggestions?

____

____

____
