# Supplementary material for: BREAst screening Tailored for HEr (BREATHE)—A study protocol on personalised risk-based breast cancer screening programme
Source: PLoS One. 2022 Mar 31;17(3):e0265965. doi: 10.1371/journal.pone.0265965 (PMC8970365; doi:10.1371/journal.pone.0265965)
Supplement: S7 Appendix — (PDF) [file pone.0265965.s007.pdf]

S7_Appendix. Obtaining appropriate threshold for the five-year absolute risk by polygenic risk score (PRS).

The Multi-Ethnic Cohort (MEC) study (part of the Singapore Population Health Studies SPHS) is supported by individual research and clinical scientist award schemes from the Singapore National Medical Research Council (NMRC, including MOH-000271-00) and the Singapore Biomedical Research Council (BMRC), the Singapore Ministry of Health (MOH), the National University of Singapore (NUS) and the Singapore National University Health System (NUHS). We thank all participants, study team and investigators for their contributions to research. Ethic approval was obtained to use the data presented (Agency for Science, Technology and Research Institution Review Board, reference number 2020-005).

The Singapore MEC study, comprising of women without breast cancer to be representative of the general population, was used to obtain reference values for the means and standard deviations of PRS. The MEC study is a population-based cohort of the general adult population of Singapore, with participants aged between 21 and 75 at enrollment (cohort described in (1). Women enrolled in MEC were ethnicity and age (+/- 5 years) matched controls to SGBCC breast cancer cases (1). Among 4,099 women were never diagnosed with breast cancer, 4,098 had genetic information (**Figure 1**). The median age was 50 years (interquartile range: 43 to 57 years).

PRS is estimated as the weighted sum of effect alleles in 313 single nucleotide polymorphisms (SNPs) found to be associated with breast cancer; using plink (version 3) with the *scoresum* option full details in Supplement Methods (2).

$$PRS = \beta_1 x_1 + \beta_2 x_2 + \cdots + \beta_k x_k + \cdots + \beta_{313} x_{313} ,$$

where x_k is the dosage of risk allele (0-2) for SNP k, β_k is the corresponding weight. The weights of the 313 SNPs for overall breast cancer risk were obtained from are of the overall breast cancer risk published by Mavaddat *et al*. (3). Each individual's PRS was standardized to the mean and standard deviation of the PRS in controls by ethnicity (Chinese, Malay or Indian in the combined dataset of MEC and MyMammo). PRS of individuals of unknown ethnicity was standardized to the mean and standard deviation of all non-breast cancer controls in MEC (n=4,098).

The five-year absolute risk was based on ethnic-specific or overall breast cancer incidence rates (period of 2013 to 2017) for Singapore Citizens, and mortality rates (year 2016) in Singapore (**Table 1)** (4, Department of 5). Both incidence and mortality rates were recorded in five-year intervals. The five-year absolute risk based on PRS was estimated using an iterative method detailed by Mavaddat *et al*. (6). In brief, an individual's PRS percentile can be obtained from the standardized PRS using *pnorm* in R. The theoretical odds ratio of this percentile as compared to the 40-60 percentile (the closest in risk to the general population) can be estimated and subsequently the corresponding five-year absolute risk can be calculated (7). The mean and standard deviations of PRS by ethnicity is presented in **Table 2**. The five-year absolute breast cancer risk by PRS percentiles is illustrated in **Figure 2**.

Figure 1. Flowchart of data used.

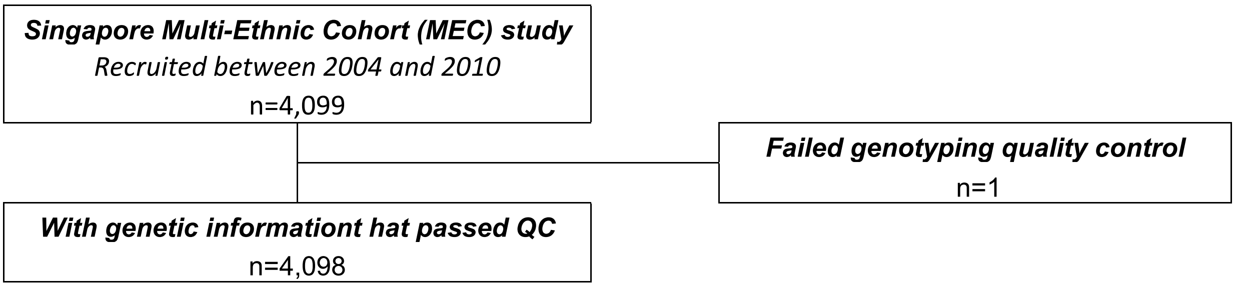

Figure 2. Five-year absolute risk of developing breast cancer, standard deviations (SD) used for the polygenic risk scores - using European weights for 313 SNP - by ethnicity, (A) all ethnicities including others, (B) Chinese, (C) Malay, and (D) Indian. Absolute risk are estimated using breast cancer incidence rates from the Singapore Cancer Registry and mortality rates obtained from the Department of Statistics, Singapore. The odds ratio used for the different percentiles can be calculated using the formula by Wen et al. equation 7 (7). The horizontal dashed line shows the five-year absolute risk of the top 1 percentile at aged 50 years.

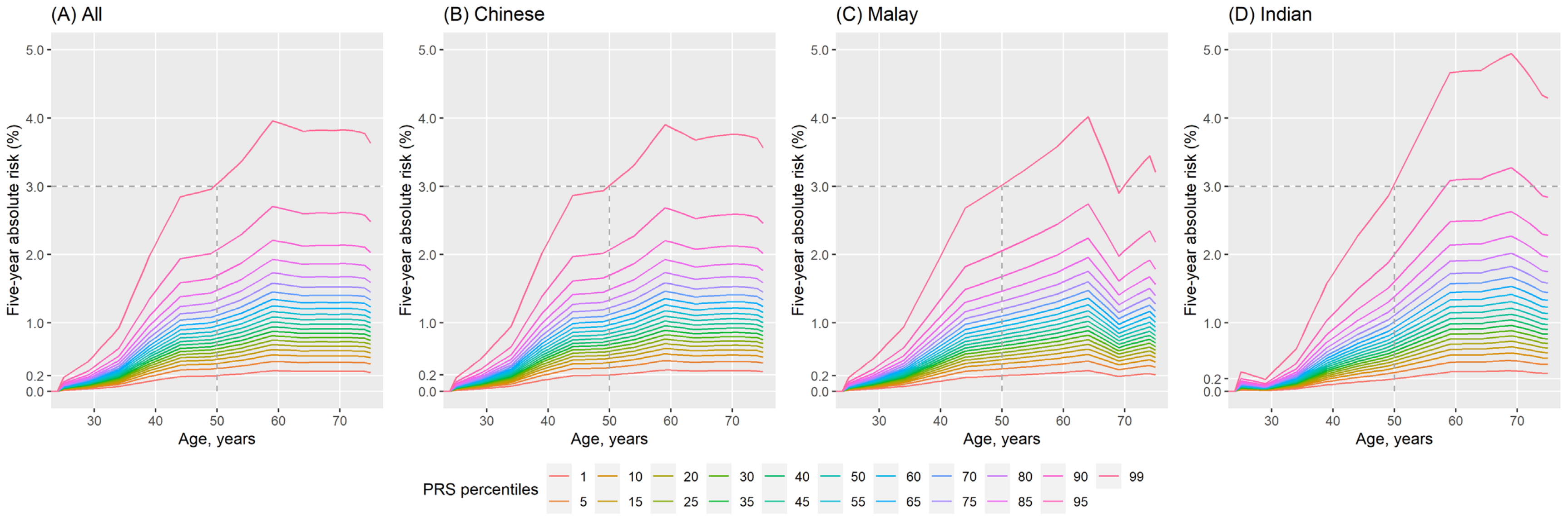

Table 1. Breast cancer incidence rates in the period 2013-2017, from the Singapore Cancer Registry, and age-specific mortality rates in 2016, from the Department of Statistics (Singapore) were used in the estimation of absolute risk (4, 5). Incidence rates are ethnicity specific while mortality rates are based on all females.

	Breast cancer incidence, per 100,000				
**Age (year)**	**All**	**Chinese**	**Malay**	**Indian**	**Mortality, per 1,000**
20	0.8	0.9	0.9	0	0.2
25	9.3	8.3	8.2	18.3	0.2
30	27.4	30.3	29.9	10	0.2
35	58.8	61.8	59.6	35.9	0.3
40	126.7	131.9	114.6	92.3	0.8
45	182.3	187.3	171	132.9	1.1
50	189.4	192	189	167.8	1.8
55	216	216.6	208.3	221.5	3
60	255	256.5	229.7	276.3	4.3
65	245.9	242.2	259.4	278.7	7
70	248.8	250	187.8	296.2	11.5
75	254.1	254.4	231.1	266.7	23.5
80	218.4	219	156.7	270.8	100
85	218.4	219	156.7	270.8	100

Table 2. Mean and standard deviation of polygenic risk scores (PRS) by ethnicity. *All includes 2 women of unknown ethnicity.

Ethnicity	N	Mean	Standard deviation
All	4,098	0.130	0.565
Chinese	3,304	0.158	0.551
Malay	574	0.109	0.571
Indian	218	-0.243	0.620

References

1. Tan KHX, Tan LWL, Sim X, Tai ES, Lee JJ, Chia KS, et al. Cohort Profile: The Singapore Multi-Ethnic Cohort (MEC) study. Int J Epidemiol. 2018;47(3):699-j.
2. Purcell S, Neale B, Todd-Brown K, Thomas L, Ferreira MA, Bender D, et al. PLINK: a tool set for whole-genome association and population-based linkage analyses. Am J Hum Genet. 2007;81(3):559-75.
3. Mavaddat N, Michailidou K, Dennis J, Lush M, Fachal L, Lee A, et al. Polygenic Risk Scores for Prediction of Breast Cancer and Breast Cancer Subtypes. Am J Hum Genet. 2019;104(1):21-34.
4. National Registry of Diseases Office N. Singapore Cancer Registry Annual Registry Report 2015. 2017.
5. Statistics Do. M810141 - Age-Specific Death Rates, Annual 2017 [updated 2020. Available from: https://www.tablebuilder.singstat.gov.sg/publicfacing/createDataTable.action?refId=13249.
6. Mavaddat N, Pharoah PD, Michailidou K, Tyrer J, Brook MN, Bolla MK, et al. Prediction of breast cancer risk based on profiling with common genetic variants. J Natl Cancer Inst. 2015;107(5).
7. Wen W, Shu XO, Guo X, Cai Q, Long J, Bolla MK, et al. Prediction of breast cancer risk based on common genetic variants in women of East Asian ancestry. Breast Cancer Res. 2016;18(1):124.
